# Multisubject Decomposition of Event-related Positivities in Cognitive Control: Tackling Age-related Changes in Reactive Control

**DOI:** 10.1007/s10548-016-0512-4

**Published:** 2016-08-13

**Authors:** Stefanie Enriquez-Geppert, Francisco Barceló

**Affiliations:** 1 Department of Clinical and Developmental Neuropsychology, University of Groningen, Groningen, Netherlands; 2 Department of Psychology, University of the Balearic Islands, Palma de Mallorca, Spain; 3 Asociación de Neuropsicologia Balear, Palma de Mallorca, Spain

**Keywords:** Cognitive aging, P300, Frontalization, Theta and beta oscillations, Task-switching, Group ICA, Functional networks

## Abstract

Age-related neurocognitive effects have been observed at different levels ranging from reduced amplitudes of even-related potentials and brain oscillations, to topography changes of brain activity. However, their association remains incompletely understood. We investigated time-frequency and time-course effects in functional networks underlying the P300 and their involvement in reactive control. Electroencephalographic (EEG) data of three different age groups (30 young: 18–26 years, 30 mid-aged: 49–58 years, 30 elderly: 65–75 years) was measured while they performed a cued colour/thickness switching task. Neural data was analysed concerning the targets. To consider restart, mixing, and switching processes, the targets´ position after a cue (first or third target) as well as their context in the single-task (distractor cue) or the mixed-task block (switch- or repeat cue) was analysed. P300 EEG data was decomposed by means of group-independent component and time-frequency analyses focusing on theta and beta oscillations. RTs generally slowed down with age (main effect group), and effects were specifically strong in targets after a switching cue (larger Cohens d). Peaking at around 300 ms, we detected five functionally independent networks reflecting the multicomponent process underlying task-switching. These networks differed in terms of their topography (parietal and frontal), their involvement in task processes (switch-specific, mixing-, restart-, and single-task processes) and in terms of frequency effects. All were affected by age, as indicated by amplitude changes of the target-P300 and power reductions most consistently shown in beta oscillations. Most extensive age-related changes were observed in one parietal network sensitive to mixing and restart processes. Changes included a topography shift, P300 and beta amplitudes, and were ongoing in the elderly group.

**Electronic supplementary material:**

The online version of this article (doi:10.1007/s10548-016-0512-4) contains supplementary material, which is available to authorized users.

## Introduction

### Declines of Executive Functions in Aging

We are currently facing periods of demographic change, which are characterized by pronounced aging due to higher life-expectancy worldwide (United Nations Development Programme 2004). While analysing the implications for health management, a crucial aspect is the cognitive decline associated with aging. Affected domains include episodic memory (Glisky et al. 2001), processing speed (Salthouse 1996), and executive functions (EFs) (Kramer et al. 1999; Bialystok 2006; Watson et al. 2010). EFs are regarded as especially imperative for success in daily life since they enable adaptive goal-oriented behaviour (Seiferth et al. 2007). In terms of aging, declines of EFs are known to reduce the success in everyday activities (Vaughan and Giovanello 2010) and to narrow the functional status in older adults (Bell-McGinty et al. 2002). Thus, it is clearly apparent that the investigation of age-related changes in executive functioning and its underlying neural mechanisms is imperative in order to face the challenges of main trends in society.

### Age-related Changes in Task Switching

EFs are defined as a bundle of higher functions controlling lower functions. Of those, motor inhibition, conflict monitoring, memory updating and task switching are the most important and most independent functions (Miyake et al. 2000; Miyake and Friedman 2010; Brydges et al. 2014; Adrover-Roig et al. 2012). Task switching requires the flexible shift between two tasks, depending of context specific cues (Rogers and Monsell 1995; Meiran 2002). Typically, the alternation between two or more tasks is investigated in the mixed-task block and compared to the single-task block without any task switching. On a behavioural level, response prolongations have been observed that are associated to at least three different processes. Most known is local switching which takes place in target processing after a cue that indicates a task switch. Local switching can be differentiated from more general mixing processes, which refer to additional demands in the context of a mixed-task compared to single-task block. A third process is related to a restart mechanism, which describes response prolongations in targets directly following a cue regardless whether a switch is require (in a mixed-task block). Concerning cognitive aging, consistent effects have been shown in mixing processes (e.g., Karayanidis et al. 2011). Aging has been suggested to lead to reduced cognitive preparation or proactive control in cue-to-target intervals and to the enhanced usage of reactive control processes (e.g., Jimura and Braver 2010). Furthermore, studies investigating adult lifespan trajectories hint to a qualitative change of task-switching at the age of 60 years. However, the neuronal changes are poorly understood. Generally, age-related cognitive decline has frequently been associated to different types of brain changes.

### Age-related Brain Changes

Concerning neurotrophic factors, which are associated to neuroprotection and to cognition as well, age-related changes have been documented (Tumati et al. 2016). On a macrostructural level, aging is associated with reduced brain weight and volume (e.g., van Petten et al. 2004). White matter integrity of axonal bundles has been shown to be decreased mostly in frontal regions (Moseley 2002; Pfefferbaum et al. 1994), and has been associated with poorer EFs performance (Grieve et al. 2007). The network implementing EFs (Niendam et al. 2012) seems to be affected similarly, as observed in grey matter declines of the highly interconnected midcingulate cortex (MCC) (Mann et al. 2011), which is considered as a network hub (Cavanagh et al. 2012). It therefore comes as no surprise that apart from structural changes, functional changes take also place in aging.

### Posterior-to-anterior Brain Activity Shifts in Aging

One of the most prominent observations in cognitive neuroscience and biopsychology is the posterior-anterior shift (PASA) (Davis et al. 2008). This shift reflects higher prefrontal activity in older participants than in younger subjects (for a review, see Grady 2012), as it was shown via functional magnetic resonance imaging (fMRI). Interestingly, the anterior shift has also been reported in task switching (e.g., Hakun et al. 2015a, b). Interestingly, with electro-encephalography (EEG), a method with high temporal resolution, age-related anterior shifts have also been observed. These include event-related potentials (ERPs), of which the P300 has received a lot of scientific attention. Although there is much debate on what the P300 exactly reflects, there is broad scientific consensus that this potentially indexes aspects of cognitive information processing. The P300 was even suggested as an index of neurocognitive aging (Polich 1996), and a joined analysis of the frontal and parietal P300 was even better in explaining behavioural performance at different points of age (van Dinteren et al. 2014). The anterior shifts of the P300 have been mostly observed in oddball tasks (e.g., Friedman et al. 2008). Crucially, concerning EFs and more precisely with task switching, age-related posterior-anterior P300 shifts have also been reported (Kopp et al. 2014; Whitson et al. 2014). However, it is important to note that EEG parameters like ERPs, which are measured at different electrode locations, reflect a composition of multiple dynamics of temporally and regionally overlapping, and functionally separable sub-processes (Arieli et al. 1996; Kiehl et al. 2005). ERPs thus mirror cumulative neural activation related to multiple processes involved in stimulus processing and evaluation (e.g., Luck 2005) which can be decomposed by time–frequency or independent component analyses.

### EEG Decomposition by Time–frequency

EEG-decomposition by time–frequency reveals synchronous processes that are supposed to coordinate neuronal spiking between and within brain circuits, namely the so-called brain oscillations that link neural activity with behaviour and thoughts (e.g., Buzsáki et al. 2013). Mainly because of anatomical constraints as slow axon conduction velocity, lower frequency bands are predestinated for neural communication between long-distance brain areas and higher frequencies for local communication (Varela et al. 2001; Buzsáki and Watson 2012). Every ERP component can thus be regarded as event-related oscillations (ERO) of a single frequency, or a superposition of multiple EROs with different frequencies. Interestingly, brain oscillations are suggested to be physiologically better interpretable than conventional ERPs, as higher accuracies are demonstrated in predicting subject’s behavioural performance (e.g., Cohen and Donner 2013).

### Age-related Time-frequency Effects

Concerning brain oscillations and aging, the scientific literature reports changes in the peak and amplitude of oscillations in the alpha (Klimesch 1999), beta- (Karrasch et al. 2004), and theta/delta bands (Kardos et al. 2014; Schmiedt-Fehr and Basar-Eroglu 2011; Cummins and Finnigan 2007). Specifically, age-related changes regarding event-related frontal-midline (fm) theta are in focus, since these oscillations have been proposed as a neural working language of EFs and are generated in the MCC (Cavanagh et al. 2012). Fm-theta oscillations have been furthermore shown to fall into the time-range of ERPs associated with EFs such as the N200/P300 complex (Huster et al. 2013; Nigbur et al. 2011; Başar-Eroglu et al. 1992). On the other hand, beta oscillations have been associated with cognition and aging as well (Karrasch et al. 2004) and might even play an important role in task-switching. As such their role has been demonstrated for active maintenance of information in working memory (Chen et al. 2016). For switching task rules, this ability seems particularly crucial, since most of all, the mixed-task block requires high amounts of active maintenance of two different sets of goal representation.

### EEG Decomposition by Independent Component Analysis

Anyhow, in order to tackle the underlying independent networks that are involved in the generation of ERPs, a further powerful tool for the decomposition of EEG data is the blind source separation by means of independent component analysis (ICA) (Bell and Sejnowski 1995). ICA solves a two-dimensional linear mixing problem of spatially and/or temporally independent sources. Applied to EEG data, ICA thus identifies functionally coherent brain networks (Hyvärinen 2013). However, the basic ICA model applies to single subject data and is limited in typical multi-subject EEG studies as it has to resolve generalization questions from single subject to a group level. While clustering techniques come with the challenge of an increased likelihood of equivocal results due to differences in the selection of algorithms, feature selection and the user bias, group-ICA seem to reflect a much more parsimonious approach to match independent components across individuals (Calhoun et al. 2009; Eichele et al. 2011; Huster et al. 2015). By applying the group-ICA approach, data containing observations from all subjects are aggregated, and independent components are estimated that are consistently expressed across subjects.

### Independent Component Analysis of the P300

In this way, Debener et al. (2005) demonstrated the involvement of different brain sources to the novelty P3, which fits to findings from intracranial, lesion and fMRI-EEG studies (Linden 2005), as well as from studies with low resolution electromagnetic tomography all pointing toward multiple neural generators underlying the P300 (Mulert et al. 2004). Regarding an attention switch paradigm, Onton et al. (2006) demonstrated that the P300 consisted of multiple underlying independent components. Hence, the application of ICA methods in EEG is a well-suited informative approach regarding the investigation of functional connectivity, tracking fast brain dynamics at different stages of processing rather than only the final end results. Especially for subjects that are known to show differences in ERPs and are therefore also expected to differ concerning the underlying components due to age-related changes, group-ICA represents an appropriate and powerful tool.

### Aim of the Study

In the current study we focused on two main issues regarding (a) the functional role of the independent networks underlying task-switching, and (b) the age-related effects in those networks with respect to two different EEG measures (P300 and event-related oscillations). For the systematic investigation of the independent functional networks, a group-ICA was performed to obtain a more complete delineation of aging effects in reactive control of switching tasks. We tracked the EEG of three different age groups, namely a young, a mid-aged and an elderly group to investigate additional later life neurocognitive changes. Participants were performing a cued visual switching task, which required the categorization of Gabor stimuli following two rules (colour and thickness). Neural data was analysed concerning targets. To consider restart, mixing, and switching processes involved in task switching, the targets´ exact position (first, third) after a cue as well as its context in the single-task block (distractor cue) or the mixed-task block (switch- and repeat cue) was analysed. Concerning the behavioural performance, we expected the known slowing effects of RTs mainly in conditions demanding EFs, as reflected in the mixed-tasks block as compared to the single-task block. Additionally, larger costs such as mixing, restart, and local switch costs were anticipated with increasing age. We expected several target- P300 independent networks, instead of only one for mainly two reasons. First, ERPs measured at the scalp surface are known to reflect cumulated neural activation related to multiple processes; and second, task-switching reflects a multicomponent process. Along with this, we expected posterior-to-anterior shifts, and age-related oscillatory changes mostly in those networks with a parietal topography. As fm-theta is regarded as neural working language of EFs, we specifically expected age-related changes in networks mostly sensitive to the switch, but also in repeat conditions. Beta power changes were similarly expected, most of all in networks sensitive to the experimental manipulations of the mixed-task block, reflecting the temporal maintenance of two task rules.

## Methods

### Participant Characteristics

30 young (20 female, mean age: 21.2 years, SD: 2.5 years), 30 mid-aged (17 female, mean age: 53.6 years, SD: 2.9 years), and 30 elderly (16 female, mean age: 68.6, SD: 2.4 years) participants took part in the present study. All participants provided a self-report regarding their neurological and psychiatric conditions. According to the Edinburgh Handedness Inventory (Oldfield 1971) all participants were right-handed apart from one who was considered to be ambi-handed. All participants had normal or corrected-to normal vision. The participants were recruited via the University of the Balearic Islands and public announcements and gave informed consent prior to study participation. In return for their participation, young participants received course credits or payment (20 Euro), while elderly received a detailed report on their cognitive results. The study was approved by the ethics committee of the University of the Balearic Islands, which is in accordance with the Declaration of Helsinki on good scientific practice. All participants were part of a larger trial examining additionally an Oddball task and a neuropsychological test battery of EFs (e.g., a Stroop test).

### Stimuli and Task Characteristics

**Fig. 1:**
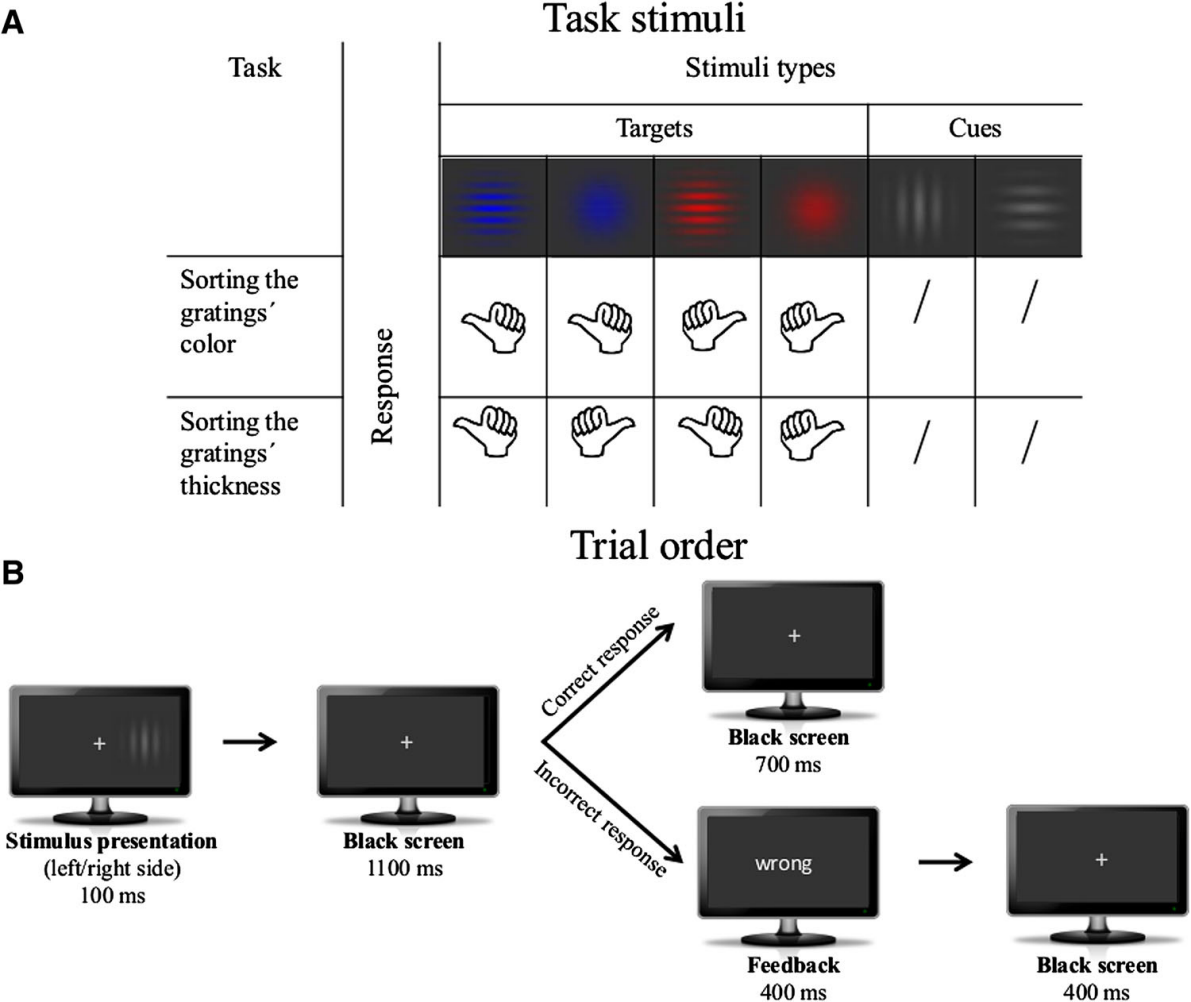
Stimuli types and trial sequence. **a** summarizes all Gabor stimuli, the two sorting tasks, as well as the corresponding responses to each condition. In the experiment two types of Gabor stimuli can be dissociated. The first stimulus type is represented by horizontally oriented targets that are either thin/thick blue or thin/thick red patches. Depending on the current task (colour sorting or gratings´ thickness sorting tasks), participants were focusing only on one characteristic (colour or thickness) and respond either with the right or left thumb. The second stimulus type was represented by grey thick Gabor gratings that were either horizontally or vertically arranged. The grey Gabor served as cues instructing the participants to continue or to switch the task. **b** depicts two possible trial types with either a total length of 1900 ms or 2000 ms. Each trial started with either a left or a right stimulus presentation for 100 ms and was followed by a black screen with a white fixation cross for 1100 ms. Within these 1200 ms the participants had time to respond (in case of a target presentation, but not for a cue stimulus). In case of a correct response, the black screen with a white cross was prolonged for 700 ms (total trial length 1900 ms). However, in case of an incorrect response, a response out of time or a false alarm, a visual feedback (“mal” for “incorrect”, “tarde” for “too late”) followed. This feedback was presented for 400 ms and the trial was completed by the black screen with the white fixation cross for further 400 ms (total trial length 2000 ms)

Participants performed a cued switching task (colour/thickness), while seated in a sound and light attenuated cabin. All task stimuli were presented on a 27-inch TFT screen with a viewing distance of 150 cm. Task presentation and performance was controlled by using Presentation (Neurobehavioral Systems). Responses were given with the thumb finger on a two button box. For the colour/thickness switching task, a variant of the intermittent-instruction paradigm (Monsell 2003) was used. Each stimulus had a visual angle of about 1° and consisted of six possible Gabor patches with either four or ten cpd thickness. There were two types of Gabor gratings, the target (blue and red Gabor gratings) and the grey cue stimuli (see Fig. 1a), which were all presented on a grey background. The target Gabor gratings were horizontally oriented and differed in colour (red vs. blue) and thickness (thin vs. thick). Each of this four combinations appeared with a probability of 21 % across the whole experiment. The two cue Gabor grating stimuli, however, differed concerning the orientation of the gratings (horizontal vs. vertical) and did not require any response. Each of the cues had a probability of 8 % across the whole experiment. Participants had to classify the target Gabor gratings concerning their colours (red = right button press vs. blue = left button press) or their thickness (thin = right button press and thick = left button press). Tasks were cued by the task-associated orientation of grey coloured Gabor patches: horizontal and vertical gratings instructed to repeat or switch the task. This cue-task assignment was counterbalanced across subjects. The experiment consisted of two blocks. First, the single-task block was presented, which is not supposed to require EFs at all. Here only the colour task had to be performed and grey Gabor gratings were to be ignored. Then, participants continued with the mixed-task block, in which the colour and thickness tasks alternated. Here they started with the colour classification task. Participants were instructed to respond as fast and as accurate as possible. Before the start of the experiment, participants received 73 test trials.

Each trial began with the presentation of a Gabor grating displayed for 100 ms, afterwards a grey screen with a fixation cross appeared for further 1100 ms (see Fig. 1b). Participants had to respond within these 1200 ms. Whenever their responses were late or wrong or whenever they responded after the cue, a visual feedback was presented for 400 ms (Spanish “mal” for “wrong” or “tarde” for “too late”), “followed by the black screen with the fixation cross for further 400 ms at the end of the trial. In case of correct responses, no feedback was presented, instead the black-screen was prolonged for 700 ms. Thus in sum, the stimulus onset asynchrony (SOA) was 1900 ms for correct and 2000 ms for incorrect trials. In the single and the mixed-tasks block each, 976 trials were presented within eight blocks which were separated by self-paced blocks. All trials were presented on a semi-randomized offline procedure to ensure that in-between two grey Gabor stimuli, four to eight coloured target Gabor gratings were presented in a sequence. All stimuli were presented either to the left or to the right of a central fixation cross and the presentation side of the stimuli was equally distributed across the whole experiment.

### EEG Recording and Preprocessing

EEG data was recorded from 60 channels using SynAmps RT amplifiers (NeuroScan, TX, USA). Electrodes were mounted on a flexible electrode cap according to the 10-10 system for electrode placement (Syncamp2 Quickcap, Compumedics, TX). In addition, the electro-oculogram (EOG) was recorded from four channels placed above and below the left eye, and on the outer canthi of both eyes. EEG and EOG recordings were taken continuously from 0.05 to 100 Hz bandpass at a sampling rate of 500 Hz. Impendences were kept below 10 kΩ. All data were referenced against the left mastoids, as ground electrode AFz was used.

For pre-processing, the MATLAB (version R2010a) based toolbox EEGLAB (Delorme and Makeig 2004, version 13.4.4b) was used. First, data was high (50 Hz) and low-pass filtered (0.5 Hz), then down-sampled to 250 Hz, and re-referenced to a common average. ICA (Runica algorithm) was used to correct for eye artefacts (Bell and Sejnowski 1995; Miyake et al. 2000). Components suggesting eye blinks or horizontal eye movements were identified by comparing the independent components (ICs) activity to the eye blink artefacts in the EOG. The corresponding topographical maps of putative ICs had to show a frontal distribution. Eye-blink related ICs were then excluded from back-projection the EEG channels. Next, data was epoched from −1600 to 1600 ms relative to stimulus onset and baseline corrected for −200 pre-stimulus to 0 ms. Based on the epoched data, a semi-automatic correction procedure was used to correct for residual artefacts. Single trials crossing a self-set threshold of 60 µV were marked automatically for visual inspection and rejection.

### Data Analysis: Behavioural Performance

To investigate the effects of aging on the behavioural performance, mean RTs and accuracies were extracted from the EEG data using MATLAB for the first and third target position after a grey cue Gabor. Further analyses were done in SPSS 15.0 (IBM Corp. Released 2013. IBM SPSS Statistics for Macintosh, Version 22.0. Armonk, NY: IBM Corp). Incorporating the position variable allows for the dissociation between local switch, mixing and restart processes associated with task-switching (see Altman 2002; Poljac et al. 2009). For the analysis of mixing and restart effects, targets of later positions are taken into account. As literature has shown that second targets positions still show “restart-effects” triggered by the cue, the third target position was selected as a reference measure in the current study. The three cost types were calculated as following: first, restart costs, calculated as the RT difference between the first and third target after a cue regardless of the cue type in the mixed-task block; second, mixing costs were calculated as RT difference between the third targets of the mixed-task block and the third targets in the single-task block; local switch costs were calculated as the difference in the mixed-task block between the first target after a switch and the first target after a repeat cue. Only correct responses within a trial run (three target trials following a grey Gabor stimulus) were taken into consideration. A 3 × 3 × 2 mixed-model ANOVA with the between group factor GROUP (young, mid-aged, elderly), and the two within-group factors TASK-RULE which refers to targets after switch-, repeat-cues or the distractor (switch, repeat, distractor) and POSITION (first, third) was set up for RTs (see Table 1). Further post hoc tests were calculated as well as effect-sizes by Cohen’s d (*M*1 - *M*2/√[(s _1_^2^ + s _2_^2^)/2). Because local switch costs were reversed and appeared as switch benefits, age-related effects were analysed by independent t-tests. Regarding the remaining costs, a 2 × 3 mixed-model ANOVA with the within-group factor COST-TYPE (restart, mixing) and the between group factor GROUP (young, mid-aged, elderly) was set up. In cases of sphericity violations, Greenhouse–Geisser corrections were performed; corrected p-values as well as ε-values are reported.

**Table 1:** Overview of the within-factor conditions. This table depicts an overview of all within-factors POSITION, which specifies the exact target position after a cue (1^st^, 3^rd^) and TASK-RULE, which instructs participants to switch or repeat the task classification (Switch, Repeat, Distractor). The between-factor GROUP (young, mid-aged, and elderly) is not depicted

	Target position		
Task-rule		1^st^	3^rd^
	Switch:	1^st^ Switch	3^rd^ Switch
	Repeat:	1^st^ Repeat	3^rd^ Repeat
	Distractor:	1^st^ Distractor	3^rd^ Distractor

### EEG Decomposition

#### Functional Connectivity Analysis: Group-ICA

To decompose EEG data into underlying functionally coherent networks, blind source separation was performed by group-ICA using the MATLAB based toolbox GIFT (Eichele et al. 2011). Single subject matrices were therefore adjusted concerning the number of trials, as the group-ICA requires the same number for each subject and condition. A minimum of 40 trials per condition were randomly selected from each subject’s available data, leading to a selection of 240 trials per subjects in total. As three subjects showed less trials (<20) in at least one of the conditions, these subjects were excluded from further analysis. For each subject, data were restricted to a two-dimensional matrix with rows corresponding to channels and columns to the time-course data.

The intrinsic data-dimensionality was estimated based on two approaches. First, a Principal Component Analysis (PCA) on a single subject level suggested a nine or ten model order, explaining >95 % of the single-subject data. Second, in order to estimate the reliability and stability of group-ICs, the group-ICA algorithm was run 100 times for each of both models using the ICASSO software (Himberg et al. 2004). This procedure revealed the 10 model order as sufficient, reliable and stable.

The group-ICA procedure itself includes two data reduction steps. On a single-subject level, data were pre-whitened and reduced by extracting most relevant and orthogonal time-courses resulting in first-level principal components that were further used as variables in a second, group-level PCA, which in turn estimated the most-relevant and orthogonal principal component time courses capturing the activity patterns correlated across subjects. In a last step, single-subject activations of the group-ICs were reconstructed by matrix multiplication of the weight matrices resulting from the group-ICA.

#### Time Frequency Analysis of Independent Components

To analyse the time-frequencies of the ICs, event-related spectral perturbations (ERSPs) were calculated for each trial per IC and subject. ERSPs represent log-transformed changes of power in dB relative to the baseline (Delorme and Makeig 2004). A Morlet-wavelet was applied with an increasing number of cycles for higher frequencies between 1 and 50 Hz, using a resolution of 150 frequency steps and 300 time point. The number of cycles started at 1 Hz and increased by 0.5 per frequency increase. The average power across the trials was divided by the frequency specific baseline values separately for each frequency to visualize power changes relative to the pre-stimulus activity.

### Statistical Analyses

#### Topography Effects of Positivities

To analyse the frontalization effects, values of the weights matrix of the four midline electrode positions were extracted for each participant. A mixed ANOVA with the in-between factor GROUP (young, mid-aged, elderly) and within factor TOPOGRAPHY (FPz, Fz, Cz and Pz) was calculated for each of the ten ICs. An age-related anterior-shift was indicated by decreased activity projections to Pz and increases to more frontal electrodes with the older participant groups. Therefore, only interaction effects of GROUP x TOPOGRAPHY will be reported.

#### Independent Component’s ERPs and ERSP

For each event-related positivity of each ICs, time values of the positivities’ peak ± 50 ms were identified based on grand-means. Regarding the sustained positivity, grand means suggested a large interval between 340 and 940 ms post stimulus. Then, mean ERPs and ERSPs were calculated across trials and extracted for each subject and condition of each IC for the above indicated positivity peak-interval. Theta (4-7.9 Hz) and beta (12.1-29.9 Hz) frequencies were taken into account. For statistical analyses of ERPs and each frequency, a mixed ANOVA with the in-between factor GROUP (young, mid-aged, elderly), and the two within factors TASK-RULE (switch, repeat, distractor), POSITION (first, third) was performed.

## Results

### Behavioural Results

#### Behavioural Performance: Reaction Times

Concerning all conditions and groups, Table 2 provides the means and standard deviations (SDs) for the RTs, while Table 3 depicts the means and SDs for all costs. As accuracies were all very high (mean across conditions: 0.93, SD: 0.09) these were considered as ceiling effects and excluded from further analysis.

**Table 2:** RTs of all conditions. This table gives an overview over all the mean RTs (and SD) of all conditions: first and third target positions after a switch, repeat, distractor cue for each group (young, mid-aged, elderly)

Group	Switch		Repeat		Distractor	
	1^st^	3^rd^	1^st^	3^rd^	1^st^	3^rd^
Young	536 ms (SD: 82 ms)	508 ms (SD: 71 ms)	569 ms (SD: 84 ms)	522 ms (SD: 69 ms)	474 ms (SD: 77 ms)	427 ms (SD: 56 ms)
Mid-aged	628 ms (SD: 62 ms)	597 ms (SD: 67 ms)	638 ms (SD: 66 ms)	611 ms (SD: 66 ms)	539 ms (SD: 60 ms)	494 ms (SD: 45 ms)
Elderly	650 ms (SD: 81 ms)	597 ms (SC: 60 ms)	658 ms (SD: 71 ms)	620 ms (SD: 58 ms)	536 ms (SD: 66 ms)	496 ms (SD: 53 ms)

**Table 3:** Behavioural costs. This figure illustrates restart, mixing costs, as well as switch benefits in ms (incl. SD) for each group (young, mid-aged, and elderly)

Group	Restart	Mixing	Local
Young	38 ms (SD: 37 ms)	87 ms (SD: 41 ms)	−32 ms (SD: 48 ms)
Mid-aged	28 ms (SD: 49 ms)	110 ms (SD: 38 ms)	−10 ms (SD: 44 ms)
Elderly	45 ms (SD: 37 ms)	112 ms (SD: 48 ms)	−8 ms (SD: 52 ms)

##### Age-independent Characteristics

Typical task-switching behaviour was statistically confirmed by a main effect of TASK-RULE (F _(1.58, 168)_ = 349, ε = .806, p < .001). As expected, the fastest RTs were shown in the single-task block (distractor > switch: t (86) = −18.4; p < .001; distractor > repeat: t (86) = −20.9; p < .001). However, RTs of repeat targets were longer than switch targets (repeat > switch: t (86) = 5.29; p < .001), thereby explaining the so-called switch benefits. A further main effect of POSITION (F _(1,84)_ = 120, p < .001) showed the expected slowing effect of RT at the first target position.

##### Age-dependent Effects

A main effect of GROUP (F (4068) = 3.32, p < .05) and a two-way interaction TASK-RULE x GROUP (F _(1,84)_ = 4.43, p < .001) were detected. A further trend towards a three-way interaction TASK-RULE x POSITION x GROUP (F _(1,84)_ = 4.43, ε = .095, p = . 053) was observed.

The younger showed fastest RTs, while RTs of both older groups showed no difference [(young < mid-aged: switch: t(56) = −5.23, p < .001, d = −1.38, effect-size r = −568; repeat: t(56) = −4.43, p < .001, d = −1.16, effect-size r = −501; distractor: t(56) = −4.274, p < .001, d = −1.12, effect-size r = −49), (young < elderly group: switch: t(56) = −5.53, p < .001, d = −1.46, effect-size r = −59; repeat: t(56) = −5.25, p < .001, d = −1.37, effect-size r = −57; distractor: t(56) = −4.05, p < .001, d = −1.06, effect-size r = −47)]. Thus, based on the effect size differences of Cohen’s d, age effects were strongest in the switch condition, followed by the repeat and finally in the distractor conditions. These differences were even enhanced between the youngest and the elderly, compared to the difference between the youngest and the mid-aged.

#### Behavioural Performance: Costs

##### Age-dependent Effects

A main effect of COST-TYPE (F_(2, 168)_ = 158.85, p < .01) was revealed. Mixing cost were significantly larger than restart costs (t(86) = 66.079, p < .001).

##### Age-dependent Effects

A further main effect of GROUP (F(2, 84) = 4.245, p < .05) showed that elderly showed higher costs than the younger (t(56) = −2.656, p < .05) and the mid-aged (t(56) = −3.336, p < .01). Switch benefits were investigated separately by independent t-tests. Here, differences were found between the young compared to both older groups (young > mid-aged: t(56) = −1.798, p < .05; young > elderly t(56) = −1.82, p < .05).

### EEG Decomposition

#### General Overview

The multi-trial multi-subject EEG decomposition resulted in ten ICs which are characterized by the event-related time-courses, ERSPs and their topographies. Time–frequency decompositions revealed sustained delta/theta activity accompanied with activity of higher frequency bands, such as beta and gamma band as reported in e.g., Enriquez-Geppert et al. (2014) and Cooper et al. (2016). The focus will be put on the target-P300 networks and only for these statistical results will be reported. Note, the P300 component result as the multiplication of the time-course times the corresponding topography of the specific IC each respectively. Five independent networks were peaking around 300 ms (IC1, IC2, IC3, IC4, IC5). Three of these were related to processes involved in the mixed-task block, that is supposed to involve EFs (IC1, IC3, IC5). Another reflected switch-specific processes (IC4) and one IC was associated with single-task processes (IC2). Table 4 summarizes the most important effects regarding the network characteristics and age-related changes and supplementary Fig. 1–3 displays an overview of the time–frequency plots of each IC for the young, mid-aged and elderly group. Importantly, all P300-networks showed age-related effects, reflected in the either the peak P300 amplitudes and/or in the time–frequency domain. Posterior-to-anterior topography shifts were observed in IC1, IC2. The most consistent time–frequency age-related effect was reduced beta power with increasing age.

**Table 4:** Overview of the network effects. The table summarizes the positivity effects of all five ICs. The first column gives the name of the network, the second the specific topography of the positivity, the third column summarizes the general sensitivity of the network concerning the experimental conditions (mixing, restart, single-task, switch-specific, position processes). In the fourth column age-related effects concerning the positivities’ amplitudes and the power of the underlying time–frequency effects are given, as well as anterior-posterior topography shifts are given

Component	Positivity	Age-independent characteristics	Age-related effects	Late age effects
IC1	Par. P300	Mixing & position	Anterior shift, ERP, β	Topography, ERP, β
IC2	Par. P300	Single-task	Anterior shift	/
IC3	Fro. P300	Restart & switch-specific	β	/
IC4	Fro. P300	Switch-specific	θ, β,	/
IC5	Fro. P300	Restart effects & switch-specific	ERP, β	/

### Independent Component 1 (IC1-P300)

**Fig. 2:**
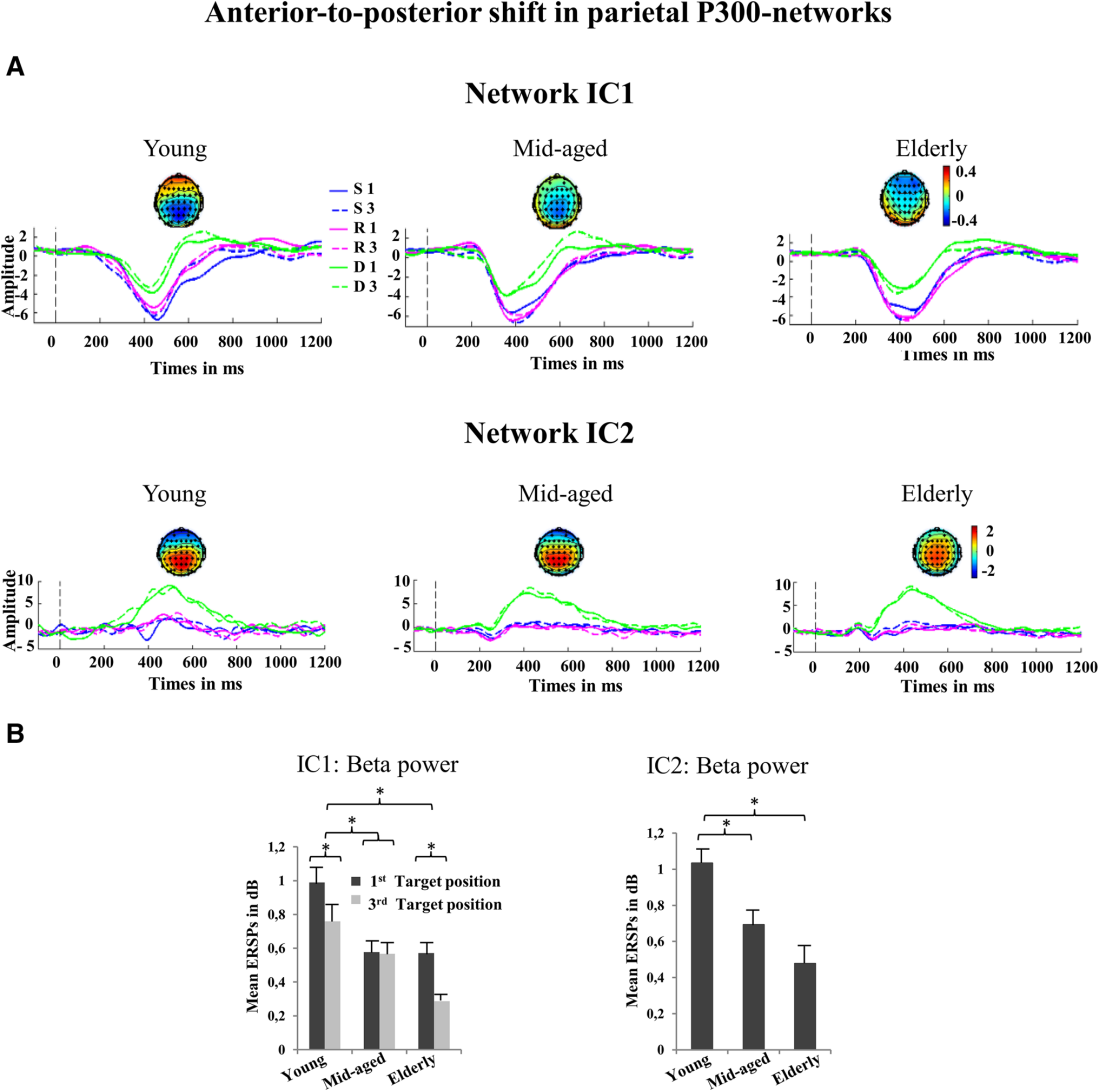
Independent component time-courses of IC1 and IC2. This figure gives an overview of the results concerning network IC1 and IC2. **a** shows the time-courses including the source topographies (negative weights are shown in blue, neutral in green and positive in red) of each IC, condition (blue line: 1^st^ Switch; dotted blue line: 3^rd^ Switch; pink line: 1^st^ Repeat; dotted pink line: 3^rd^ Repeat; green line: 1^st^ Distractor; dotted green line: 3^rd^ Distractor), and group (young, mid-aged, elderly), respectively. For IC1 and IC2, two networks with a parietal oriented topography a posterior-to-anterior shift can be observed with increasing age. For the interpretation of the polarity of the IC-ERPs, the weight topography matrix has to be multiplied with the time-course of the respective IC. Therefore, both IC1 and IC2 reflect positivities peaking at about 400 ms post stimulus (IC1-P300, IC2-P300). **b** shows (* = statistically significant) differences regarding the power of beta oscillations during the positivities (and standard errors of the mean). As can be seen, young participants show stronger beta power than both older groups in both networks

This component exhibits a later P300 at about 400 ms (IC1-P300) with a parietal maximum in topography. Here extensive age-related effects with further later life neurocognitive changes are found. A strong frontalization effect is observed in both older groups (see Fig. 2) and further age-related effects are observed concerning the P300 and oscillations (see Table 4 for an overview).

#### Age-independent Characteristics of IC1-P300

##### IC1-P300 Time-course Effects

First of all, main effect of TASK-RULE (F(2, 168) = 55.081, p < .001) was revealed. Generally, amplitudes were strongly increased regarding targets of the mixed-task compared to the single-task block (switch > distractors: t(86) = −8.321, p < .001; repeat > distractor: t(86) = −7.745, p < .001). A second main of effect of POSITION (F(1,84) = 10.182, p < .001) showed enhanced amplitudes at the first compared to those at the third target position (t(86) = –2.924, p < .01) (see Fig. 2a).

##### IC1-P300 Time Frequency Effects

Concerning theta oscillations, a main effect of TASK-RULE (F(2, 168) = 5.08, p < .01) was detected. Post-hoc effects revealed that this effect was driven by larger amplitudes in switch and distractor conditions compared to the repeat condition (switch > repeat: t(86) = 3.31; p < .01; distractor > repeat: t(86) = 2.52; p < .05). Regarding beta oscillations, a main effect of POSITION (F(2, 84) = 11.96, p < .01) was shown (see Fig. 2b). Stronger beta oscillations were observed in the first compared to the third target position (t(86) = 3.4; p < .01).

#### Age-dependent Effects of IC1-P300

##### IC1-P300 Age-related Frontalization Effects

An interaction of GROUP x TOPOGRAPHY (F (6, 252) = 7.55, p < .001) was shown for the IC1-P300 topography. As expected, the young group showed stronger Pz activity than the elderly (t(56) = −3.37; p < .01), who showed a reversed topography pattern at frontal electrode positions (elderly > younger: Fz: t(56) = 4.94, p < .001; FPz: t(56) = 5.21, p < .001). Interestingly, the mid-aged showed stronger Fz activity compared to the younger (Fz: t(56) = 2.592, p < .05; FPz: t(56) = 2.597, p < .05), while they did not differ concerning Pz. Compared to the elderly, mid-aged revealed stronger Pz activity (t(56) = −2.03, p < .05).

##### IC1-P300 Age-related Time-course Effects

IC1-P300 was strongly affected by age as shown by the main effect of GROUP (F(2,84) = 18.448, p < .001). Increased amplitudes were detected in both older group compared to the young group (mid-aged > young: t(56) = 5.7, p < . 001; elderly > young: t(56) = 3.47, p < . 001). However, the mid-aged group showed also enhanced amplitudes than the younger group (t(56) = 2.67, p < . 001). Two further interactions were revealed (TASK-RULE x GROUP: F(4,168) = 3.481, p < .0; POSITION x GROUP interaction were detected: F(2,84) = 9.192, p < .001). Post-doc revealed that only in the mid-aged group first target positions elicited the largest amplitudes compared to the third target position (t(28) = -3.992, p < .001).

##### IC1-P300 Age-related Time–frequency Effects

With regard to beta effects, a main effect of GROUP (F(2, 84) = 5.858, p < .01) was revealed. The young group showed higher beta power than the mid-aged (t(56) = 3.112, p < .01) and elderly (t(56) = 3.112, p < .01). A further interaction of GROUP x POSITION (F(2, 84) = 4.881, p < .05) demonstrated, that the position effect was only observed in the younger (t(28) = 2.24, p < .05) and the elderly (t(28) = 3.257, p < .01), not in the mid-aged (see Fig. 2b).

### Independent Component 2 (IC2-P300)

This component depicts a positivity at about 400 ms (IC2-P300) that is augmented by targets in the single-task block. Its topography exhibits a parietal maximum. Thus, this component also resembles the traditional P3b label. However, the topography is also frontalized in both older groups. In general, age-related effects are rather weaker than in IC1-P300, and no differences are found between both older groups (see Fig. 2).

#### Age-independent Characteristics of IC2-P300

##### IC2-P300 Time Course Effects

A main effect of TASK-RULE (F(2, 168) = 336.334, p < .001) was revealed. Largest amplitude effects were seen for targets after distractors in the single-task block (distractor > switch (t(86) = −10.35, p < .001; distractor > repeat (t(86) = −11.37, p < .001). The other conditions did not elicit a positivity at all.

##### IC2-P300 Time–frequency Effects

Regarding theta oscillations an interaction of TASK-RULE x POSITION (F(2, 168) = 4.992, p < .01) was revealed.

#### Age-dependent Effects of IC2-P300

##### IC2-P300 age-related frontalization effects

Here, an interaction of GROUP x TOPOGRAPHY (F(6, 252) = 3.803, p < .001) was confirmed. As expected, young participants showed larger Pz amplitudes than the elderly (t(56) = 2.657, p < .05). At frontal electrode positions (FPz, Fz), amplitudes were enhanced for the elderly compared to the younger (FPz: t(56) = −2.642, p < .05; Fz: t(56) = −3.283, p < .01). However, trends reflecting slight differences between the mid-aged and elderly were apparent at frontal electrodes (FPz: t(56) = −1.9, p = .063; Fz: t(56) = −1.8, p = .077).

### Independent Component 3 (IC3-P300)

**Fig. 3:**
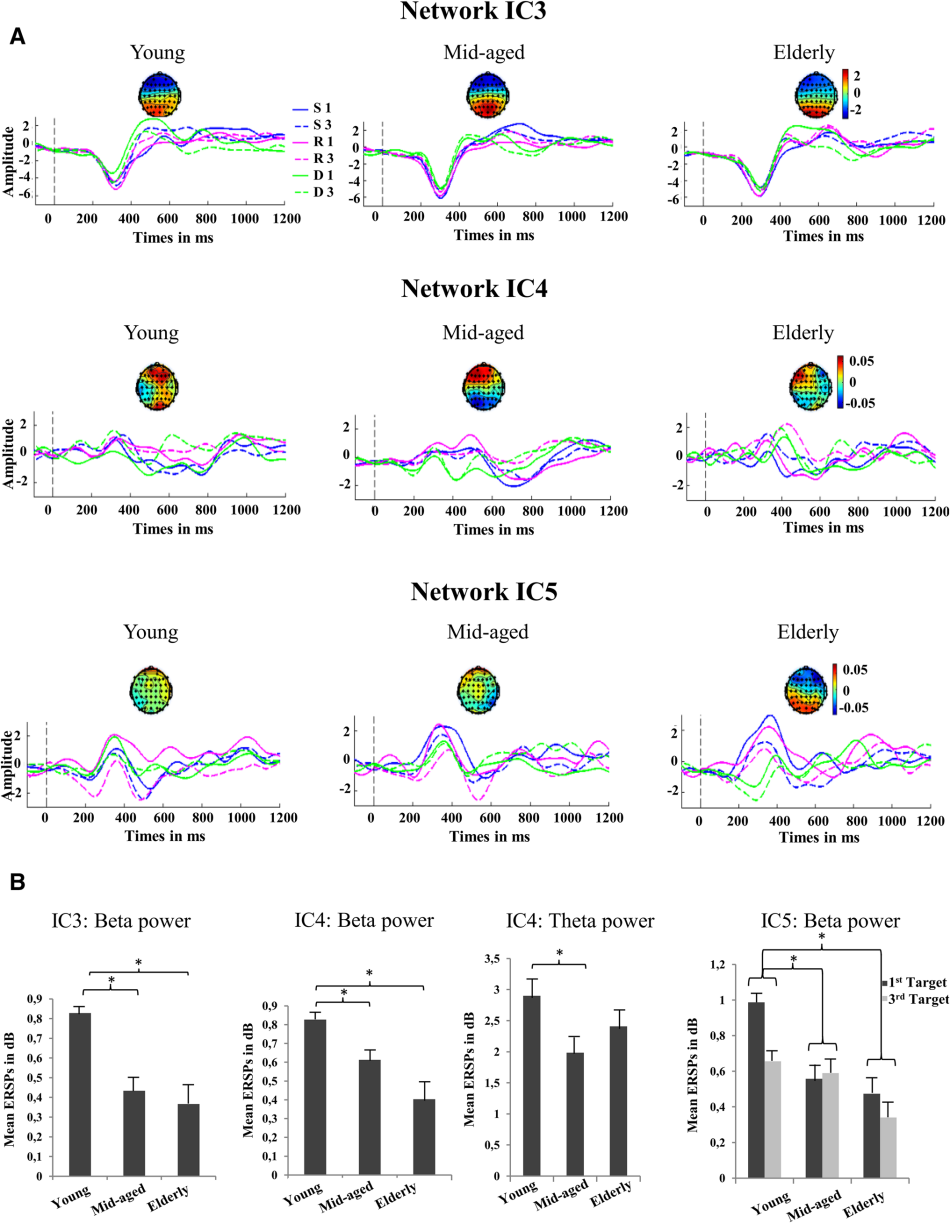
Independent component time-courses of IC3, IC4 and IC5. This figure presents an overview of the results concerning network IC3, IC4and IC5. **a**, **c** and **d** show the time-courses including the source topographies (negative weights are shown in blue, neutral in green and positive in red) of each IC, condition (blue line: 1st Switch; dotted blue line: 3rd Switch; pink line: 1st Repeat; dotted pink line: 3rd Repeat; green line: 1st Distractor; dotted green line: 3rd Distractor), and group (young, mid-aged, elderly), respectively. Only IC4 and IC5 depict age-related topography shifts (IC4: increased central activity with age; IC5 central shift with age). Note, for back reconstruction to the electrode space, the weight topography matrix has to be multiplied with the time-course of the respective IC. Therefore, the components reflect positivities peaking at about 300 ms post stimulus (frontal IC3-P300, frontal IC4-P300). **b** and shows (* = statistical significant) differences regarding the power of beta oscillations in all three networks (and standard errors of the mean). Here again, young participants demonstrate stronger beta power than both older groups. Additionally, an age-related difference in theta power can be seen for IC4, young participants elicit stronger theta power in the first target position after switch cues than mid-aged

IC3-P300 exhibits a positivity at about 300 ms (IC3-P300), which is sensitive to the target position in the mixed-task block and has a frontal age-independent topography (see Fig. 3).

#### Age-independent Characteristics of IC3-P300

##### IC3-P300 Time-course Effects

Two main effects of TASK-RULE (F(2,168) = 11.717, p < .001) and POSITION (F(2,168) = 11.613, p < .001), as well as an interaction of TASK-RULE x POSITION (F(2,168) = 8.03, p < .001) were found. Higher amplitudes of the first position targets compared to the third were observed in the mixed-target block only (1^st^ switch > 3^rd^ switch: t(86) = −3.737, p < .001; 1^st^ repeat > 3^rd^ repeat: t(86) = −3.75, p < .001).

##### IC3-P300 Time–frequency Effect

Regarding theta, two main effects of TASK-RULE (F(2, 168) = 3.79, p < . 05), POSITION (F(2,168) = 5.19, p < .05), as well as an interaction of TASK-RULE x POSITION (F(2,168) = 5.268, p < .01) were shown. Post-hoc tests revealed enhanced theta-related activity in the first target position after a switch cue compared to the third position after a switch (t(86) = 3.84; p < .01), and in comparison to the first target after a repeat cue (1^st^ switch > 1^st^ repeat: t(86) = 4.201; p < .01).

#### Age-dependent Effects of IC3-P300

##### IC3-P300 Age-related Time–frequency Effect

Beta oscillations demonstrated a the main effect of GROUP (F(2, 84) = 6.031, p < .01). Younger participants elicited higher beta power compared to the mid-aged (t(56) = −3.377, p < .001) and the elderly participants (t(56) = 3.122, p < .01).

### Independent Component 4 (IC4-P300)

This component depicts a relatively early positivity at about 300 ms (IC4-P300) with an age-independent frontal topography (see Fig. 3). IC4-P300 shows switch-specific behaviour and age-related effects in the theta time–frequency domain.

#### Age-independent Characteristics of IC4-P300

##### IC4-P300 Time–frequency Effects

An interaction of TASK-RULE x POSITION (F(2,168) = 3.933, p < .05) was revealed in theta oscillations. Post-hoc effects showed enhanced theta oscillations of first target position after a switch compared to the third position (1st switch > 3rd switch: t(86) = 2.353, p < .05) and also increased theta power of the first position targets after a switch cue compared to those after repeat (1st switch > 1st repeat: t(86) = 3.56, p < .01). A further main effect of POSITION (F(1,84) = 14.507, p < .001) was found regarding beta oscillations. First position targets elicited stronger beta oscillations than third target positions (t(86) = 3.79, p < .01).

#### Age-dependent Effects of IC4-P300

##### IC4-P300 Age-related Time–frequency Effects

Age-related effects were revealed in theta oscillations. Planned comparisons revealed expected higher theta power of younger participants compared to mid-aged participants when they had to switch tasks (S1 effects: t(56) = 2.735, p < .01). However, also beta oscillations revealed a main effect of GROUP (F(2, 84) = 5.835, p < .01) (see Fig. 3b). Younger elicited higher beta power than both the mid-aged (t(56) = 2.142, p < .05) and elderly (t(56) = 3.178, p < .01).

### Independent Component 5 (IC5-P300)

This component reflects a further positivity at 400 ms (IC5-P300) related to position effects of the repeat condition with an age-independent weak central topography (see Fig. 3). Age-dependent changes are seen in beta oscillations between the young and both older groups.

#### Age-independent Characteristics of IC5-P300

##### IC5-P300 Time-course Effects

Here, a main effect of POSITION (F(1, 84) = 11.07, p < .001). First position targets elicited a P300 at all (t(86) = 2.982, p < .01). However, an interaction of TASK-RULE x POSITION (F(2,164) = 5.452, p < .01) was also detected and post hoc tests revealed, that the position effects was mainly driven by differences between repeat targets (1^st^ repeat vs 3^rd^ repeat: t(86) = 4.3, p < .001).

##### IC5-P300 Time–frequency Effects

An interaction of TASK-RULE x POSITION was observed in the theta (F(1, 84) = 3.588, p < .05). Post-hoc tests revealed enhanced theta-related activity in the first target position after a switch cue compared to the third position after a switch (t(86) = 2.98; p < .01), and in comparison to the first target after a repeat cue (1^st^ switch > 1^st^ repeat: t(86) = 2.74; p < .01). Two further main effects in beta oscillations were found (TASK-RULE: F(2, 168) = 4.681, p < .05 and POSITION: F(2,168) = 9.194, p < .01). First position targets elicited stronger beta oscillations than third target positions (t(86) = 2.9, p < .01), and beta in switch conditions elicited higher power than in repeat conditions (t(86) = 3.54, p < .01).

#### Age-dependent Effects of IC5-P300

##### IC5-P300 Age-related Time-course Effects

Only a trend of TASK-RULE x GROUP (F(4, 168) = 2.138, p = .078) was detected.

##### IC5-P300 Age-related Time–frequency Effects

Regarding beta oscillations, a main effect of GROUP (F(2, 84) = 5.858, p < .01) with the typical effect of higher beta power in young compared to the mid-aged (t(56) = 2.481, p < .05) and elderly (t(56) 3.327, p < .01) revealed. A further interaction of POSITION x GROUP (F(2, 84) = 4.881, p < .05) was found. Differences regarding the target position were only observed in the mid-aged group (t(86) = 2.98, p < .01).

## Discussion

The current study extends previous findings on reactive control in task switching and aging by drawing a critical line between target-P300, topographies (age related anterior shifts), and event-related oscillations (theta and beta) in functionally independent neural networks. Concerning the general behavioural performance, the expected step-wise decline in aging was observed most pronounced in conditions that require EFs. Mixing and restart costs and switch benefits were all similarly affected by age. Regarding the EEG decomposition three main findings are worthy to be emphasized. As first main result, a total of five functionally independent networks, peaking around 300 ms were identified by group-ICA (IC1–IC5). Thus, these results confirm the multicomponent process underlying task-switching. The second main finding reflects widespread age-related neural changes. All networks were affected by age, although to a different amount of complexity. The third main result, hints to ongoing changes in later life. Although the most pronounced behavioural and neural differences occurred between the young and both older groups, further alterations were seen between mid-aged and older participants (e.g., effect sizes of RT-differences, as well as switch benefits) and in neural processing. In the following, these three main findings will be discussed in detail.

### Multicomponent Process in Task-switching

The EEG decomposition shows that the P300 is composed of several components with a neuronal rich landscape and differential involvement in task processes. Regarding the functional role of the networks, one P300 network was switch-specific (IC4-P300) and a further three networks were involved in processes related to the mixed-task block (IC1-P300, IC3-P300, IC5-P300). Thus, these networks are all considered to reflect different aspects of executive functioning. The remaining P300 network showed sensitivity to single-task processes (IC2-P300), thus reflecting basic cognition. The switch-specific network IC4-P300, regarding both its topography and its peak latency, seemed to match criteria for the definition as a classical P3a. Interestingly, effects were only shown in the task-frequency domain with expected increased effects in fm-theta oscillations. The IC1-P300 network in contrast, peaked at a later latency with a more parietal scalp distribution, and hence, it rather fits with the traditional P3b. IC1-P300 is highly involved in processes of the mixed-block and showed further sensitivity to the position of targets as effects in the time-course and time–frequency domain showed. Therefore, this network might reflect complex and control demanding processes, when confronted with bivalent stimuli and task uncertainty (e.g., Cooper et al. 2016). Such a function might be related to the concept of competition management (Rubin and Meiran 2005), in task-switching and may reflect global or sustained control mechanisms (e.g., Braver and Barch 2002). In contrast, IC3-P300 resembled also the P3a –although differently than in IC4-P300– and may reflect independent functional processes of task-interference and the reactivation of task-sets (e.g., Altman 2002) associated to restart processes in the time-course domain and in the theta band. Very similar effects were shown in IC5-P300, a further type of P3a-like component network showing larger activation on first target positions, reflecting on the higher cognitive demands following informative and unexpected cueing events (Barceló & Cooper, in press). IC2-P300 is a component peaking late and parietally (P3b). This network is functionally involved in the execution of a single simple and nearly automatic task triggered by univalent stimuli. Regarding the effects of age on neural functioning, widespread changes are reflected in all networks and affecting different measurements of EEG and fitting to the qualitative change in task-switching processing at the age of 60 (see Karayanidis et al. 2011).

### Widespread Age-related Changes

Most extensive and complex age-related changes were found in IC1-P300 that led to posterior-to-anterior topography shifts, P300 amplitude variations, and beta power reductions. Regarding the remaining networks less complex age effects were revealed (IC2: posterior-to-anterior amplitude shift similar to IC1; IC3: beta power reductions with age; IC5: P300-amplitude & beta power reductions with age; IC4: theta and beta power changes).

### Age-related Switch-specific Effects in Theta

General effects in theta oscillatory activity was related to switch processes of the switch-specific component IC3-P300, reflecting rather the transient control processes of EFs (see Braver and Barch 2002). As expected these were affected by age as shown by reduced amplitudes. At a behavioural level these effects were not accompanied by increased switching costs but by reduced switching benefits. However, the processes behind such benefits are less clear less familiar, although already reported earlier in literature (e.g., Cherkasova et al. 2002; Barton et al. 2006, both studies utilized SOAs of 2000 ms). It is a well-known fact, that the increase of SOA reduces switching cost as presented with long SOAs (e.g., 900 ms in Logan and Bundesen 2003). Such cost reductions could be expected in the current design that even includes SOAs of 1900 ms. Among others, Hunt and Klein (2002) used a cued pro- and anti-saccadic switching task including long SOAs (200, 500, and 1100 ms) and reported switch costs with the shortest SOA, the vanishing of switch costs in the 500 ms SOA condition, and switch benefits with the longest SOA. According to this, they brought up the idea of alertness drifts as a possible explanation for switch benefits. With long SOAs, task reconfigurations may at least be mostly completed before target presentation. In the repeat condition the renewed implementation of the same task set would be faster than in the case of switching. This would lead to an increased likelihood to drift alertness during the long cue-target interval of this experimental condition, and could subsequently end up in increased RTs at the time of the actual target presentation. In contrast, longer lasting task set reconfigurations during the cue-target interval in the switch condition may result in a reduced chance of alertness drifts, which would appear as switch RT benefits when subtracting switch minus repeat trials. Such an explanation seems plausible, but is still rather speculative; anyhow, and regardless of the exact source for switching benefits, this phenomenon has been shown to be age-sensitive. Reduced switching benefits in both older groups are possible due to consequences of age-related slowing on reduced alertness drifts when repeating the same task-set. However, a further observation that emerges from the EEG decomposition into EROs is the general age-related beta power reductions nearly in all networks.

### Age-related Time-frequency Effects in Beta

The functional role of beta in this study is reflected as a very systematic target position effect independent from the task context (single-task vs. mixing-task block) seen across networks. Thus, it might be involved in interruptions of regular task-responses by cues, similar to the idea of Gopher et al. (2000) who suggested task-rhythm to play a role in task-switching. Increased beta oscillations in the first position compared to the third, resemble the beta-bursts reported in Cooper et al. (2016), however with age, beta oscillations were generally reduced. The fact, that these age-related beta power reductions were found across most functional networks, fits to findings which show that beta oscillations are observed all over the brain (Uhlhaas, et al. 2008) in all cortical areas and numerous subcortical structures (Uhlhaas and Singer 2013). Its generation has been linked to Glutamate, NMDA receptor-, and GABAa receptor activity (Traub et al. 2004; Yamawaki et al. 2008). In general, the functional role of beta is less analysed compared to other frequency bands (see review Huster et al. 2013). Beta oscillations have primarily been associated with somatosensory and motor functions (Pfurtscheller, et al. 1996; Pfurtscheller and Klimesch 1991, review Kilavik et al. 2013). However, concerning the functional role in cognition, beta oscillations have also been linked to working memory, working memory load (Pesonen, et al. 2006; Pesonen et al. 2007), and in maintenance in working memory (Chen et al. 2016), as well as to attention and cognitive control (Stoll et al. 2015). Among others, Tallon-Baudry et al. (2004)??? demonstrated how large coordination of distributed neural activity in two sites over the posterior infero-temporo cortex is synchronized in the beta frequency range during correct trials, which require working memory, whereas synchrony failed with incorrect responses. Similarly, long-range synchronization and beta-band activity has been shown in attention control (Schnitzler and Gross 2005). Transient long-range phase synchronization in the beta-band has been interpreted as communication within the fronto-parieto-temporal attention network. Interestingly, healthy aging has been related to oscillatory beta responses in working memory (e.g., Karrasch et al. 2004) and to attention, as shown by reduced beta power associated with reduced behavioural performance in older compared younger people (Gola et al. 2012). Pathological aging, nevertheless, stands also in connection with decreased beta power. Missionnier and colleagues (2007) reported, for example, a continued reduction of beta power in progressive mild cognitive impairment as well as in Alzheimer´s disease observed in a two-back task compared to healthy elderly. Along the same line, Kurimoto et al. (Kurimoto et al. 2012) reported reduced beta synchrony in the right central area of patients with Alzheimer’s disease during a Sternberg’s visual memory task. Hence, the current results add to the growing body of evidence that age-related beta power reductions are not only observable in Sternberg-, n-back- and detection tasks, but also in more complex switching tasks.

### Age-related Posterior-to-anterior Shifts

A further interesting finding of the current study is the age-related posterior-to-anterior shift which is observed in the two parietal networks (IC1-P300 and IC2-P300). In view of the fact that age-related topography shifts are primarily observed in Oddball tasks (e.g., Juckel et al. 2012; O’Connell et al. 2012) both with EEG and fMRI, whereas in complex switching tasks this phenomenon has only been recently assessed with fMRI (Hakun et al. 2015a, b), the electroencephalographic investigation in this study regarding IC1-P300 provides insightful findings into the timing and type of oscillatory communication and marks on this way a necessary next step. One of the few studies combining corresponding methods as fMRI and diffusion tensor imaging (DTI), have brought into play mechanisms such as reduced inter-hemispheric prefrontal signalling. For instance, (Zhu et al. 2015) studied task switching with fMRI-DTI in a cross-sectional study. Thereby they observed lower integrity of white matter paths connecting frontal brain structures, which in turn reflected enhanced activity in elderly compared to younger participants. In a longitudinal study with elderly, Hakun and colleagues (Hakun et al. 2015a) measured fMRI-DTI even twice. At the second measurement after 3 years, they reported increased activations in the prefrontal cortex (PFC) during task switching compared to the first measurement. These changes were furthermore associated with declines of white matter integrity in the corpus callosum, the fibre bundle connecting the PFC of both hemispheres. Interestingly, increased activity in the left ventro-lateral PFC was associated with increased response latencies. Thus, such PFC activity changes have been interpreted as functional attempts to overcome structural declines underlying brain communication. Concerning the interpretation of these observations, different concepts emerged, such as the compensation-related utilization of neural circuits hypothesis (CRUNCH), and the scaffolding theory of aging and cognition (STAC) (Reuter-Lorenz and Park 2010. However, dysfunctional accounts point to declines in regional functional specificity and age-related reductions in efficiency (Rypma and D’Esposito 2000).

### One-going Changes in Later Life

Interestingly, the observed effects in both networks, IC1 and IC2, may hint to a different trajectory of neural changes during aging regarding the topography shifts and oscillatory power changes. With regard to the EF-related network IC1, the PASA is revealed in the mid-aged by increased frontal activity, which is observed additionally to the pronounced posterior activity. Further topography changes are visible in the elderly, the former posterior activity is considerably reduced and a marked frontal topography evolved. In contrast, the anterior-shift to a central topography maximum in IC2, which reflects general cognitive processes, might evolve later, as these deviations were only apparent between the youngest and oldest group. With regard to the oscillatory changes, beta power reductions seem to rather emerge earlier in life and especially before 50 years of age in both networks IC1 and IC2, since a marked divide is already evident between the young and the mid-aged group, whereas both older groups cannot be distinguished. Thus, in this cross-sectional study, reduced neural communication in the beta frequency range is observed before other topographical reorganizations in the subsequent course of development. These two effects combined might contribute to less efficient neuronal processing in advanced aging.

### Conclusions and Outlook

To conclude, the results of the multi-subject decomposition of ERPs in task switching open new prospects in understanding aging in reactive control, but comprise also several implications for future studies related to basic and translational research. A next important step might be the understanding of the associations between functional P300-networks and age-related anatomical changes, similar to Zuh et al. (2015), who combined functional with anatomical data. Interestingly, the usage of DTI (Giorgio et al. 2010) revealed widespread decrease of white matter microstructure from young adulthood on, including posterior regions such as the splenium of the corpus callosum and the posterior limb of the internal capsule. As it was shown for gamma band activity and the corpus callosum, better myelinated pathways facilitate efficient inter-hemispheric information transfer (Zaehle and Herrmann 2011). Hence, it yet remains to be investigated whether the integrity of abnormal signal transmission may also be a neuroanatomical prerequisite for efficient synchronization for theta and beta oscillations in general, and further, what specific pathways are associated with oscillations in the networks underlying set-shifting.

The current results might furthermore offer a starting point for the application of scientific knowledge into health benefits. Although cognitive declines and age-related neural changes are known in older adults, current concepts as the life span theory propose that cognitive enhancement is possible throughout the whole life span (Baltes et al. 2006). The decomposition of EEG into independent neural networks and the analyses of relevant ERPs and EROs, as well as the assessment of brain-behaviour relations provide specific neural targets for neuroscientific brain trainings, such as neurostimulation or—feedback, in order to maintain and enhance set-shifting in the elderly.

## Electronic supplementary material

Below is the link to the electronic supplementary material.

Supplementary Figure 1. Time-frequency plots of IC1 and IC2. Shown are the time-frequency plots (ERSP) of all conditions (S1: 1st Switch; S3: 3rd Switch; R1: 1st Repeat; R3: 3rd Repeat; D1: 1st Distractor; D3: 3rd Distractor) for the specific group and for all frequency bands (delta, beta, theta, alpha, low- and high gamma) for all age groups (young, mid-aged, elderly) of each component respectively. (TIFF 11895 kb)

Supplementary Figure 2. Independent component features of IC3 and IC4. Shown are the time-frequency plots (ERSP) of all conditions (S1: 1st Switch; S3: 3rd Switch; R1: 1st Repeat; R3: 3rd Repeat; D1: 1st Distractor; D3: 3rd Distractor) for the specific group and for all frequency bands (delta, beta, theta, alpha, low- and high gamma) for all age groups (young, mid-aged, elderly) of each component respectively. (TIFF 11510 kb)

Supplementary Figure 3. Independent component features of IC5. Shown are the time-frequency plots (ERSP) of all conditions (S1: 1st Switch; S3: 3rd Switch; R1: 1st Repeat; R3: 3rd Repeat; D1: 1st Distractor; D3: 3rd Distractor) for the specific group and for all frequency bands (delta, beta, theta, alpha, low- and high gamma) for all age groups (young, mid-aged, elderly) of IC5. (TIFF 16348 kb)
