# Supplementary figures and images for: Multisubject Decomposition of Event-related Positivities in Cognitive Control: Tackling Age-related Changes in Reactive Control
Source: Brain Topogr. 2016 Aug 13;31(1):17–34. doi: 10.1007/s10548-016-0512-4 (PMC5772116; doi:10.1007/s10548-016-0512-4)

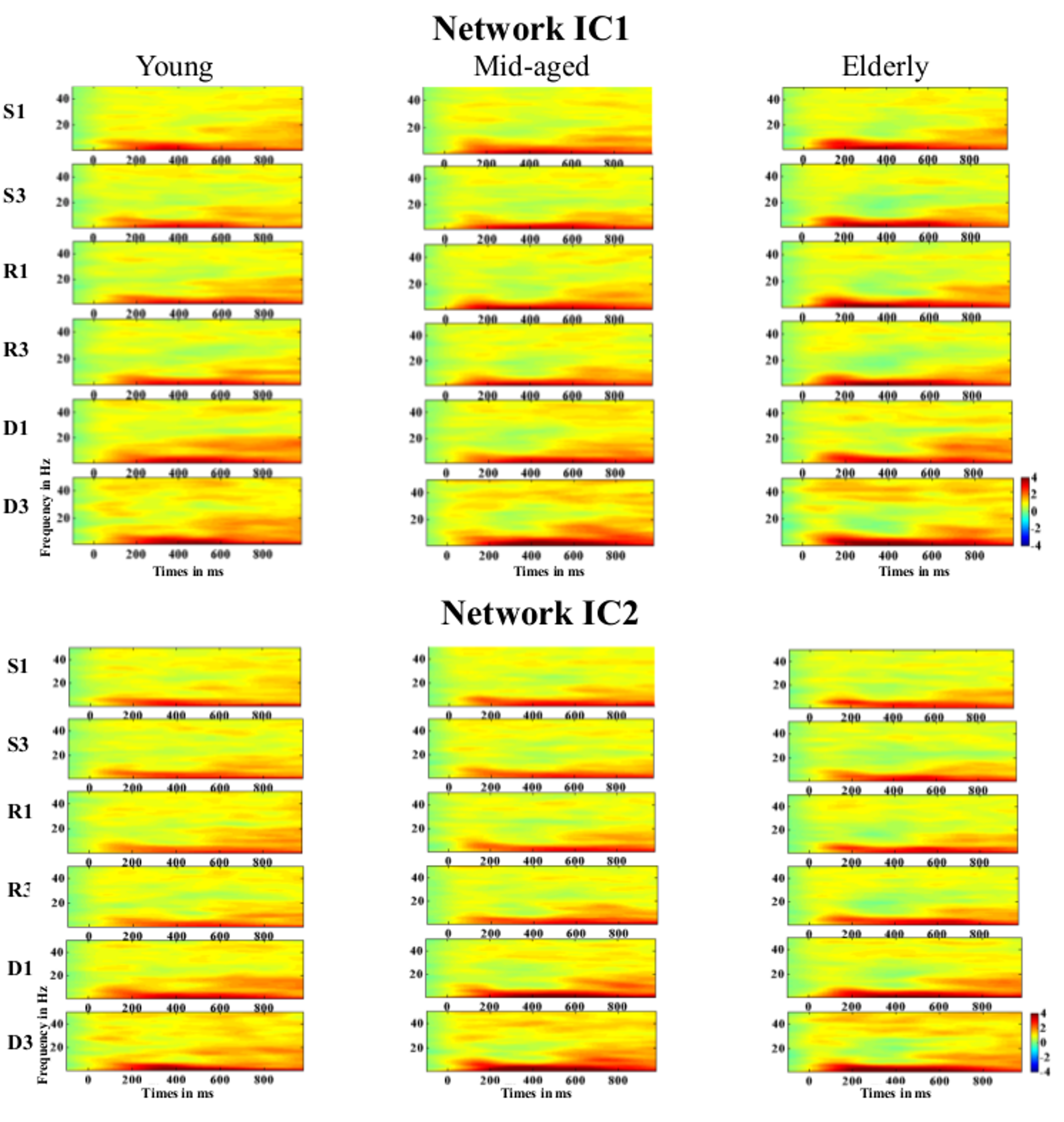

Supplement: Supplementary file 1 — Supplementary Figure 1. Time-frequency plots of IC1 and IC2. Shown are the time-frequency plots (ERSP) of all conditions (S1: 1st Switch; S3: 3rd Switch; R1: 1st Repeat; R3: 3rd Repeat; D1: 1st Distractor; D3: 3rd Distractor) for the specific group and for all frequency bands (delta, beta, theta, alpha, low- and high gamma) for all age groups (young, mid-aged, elderly) of each component respectively. (TIFF 11895 kb) [file 10548_2016_512_MOESM1_ESM.tif]

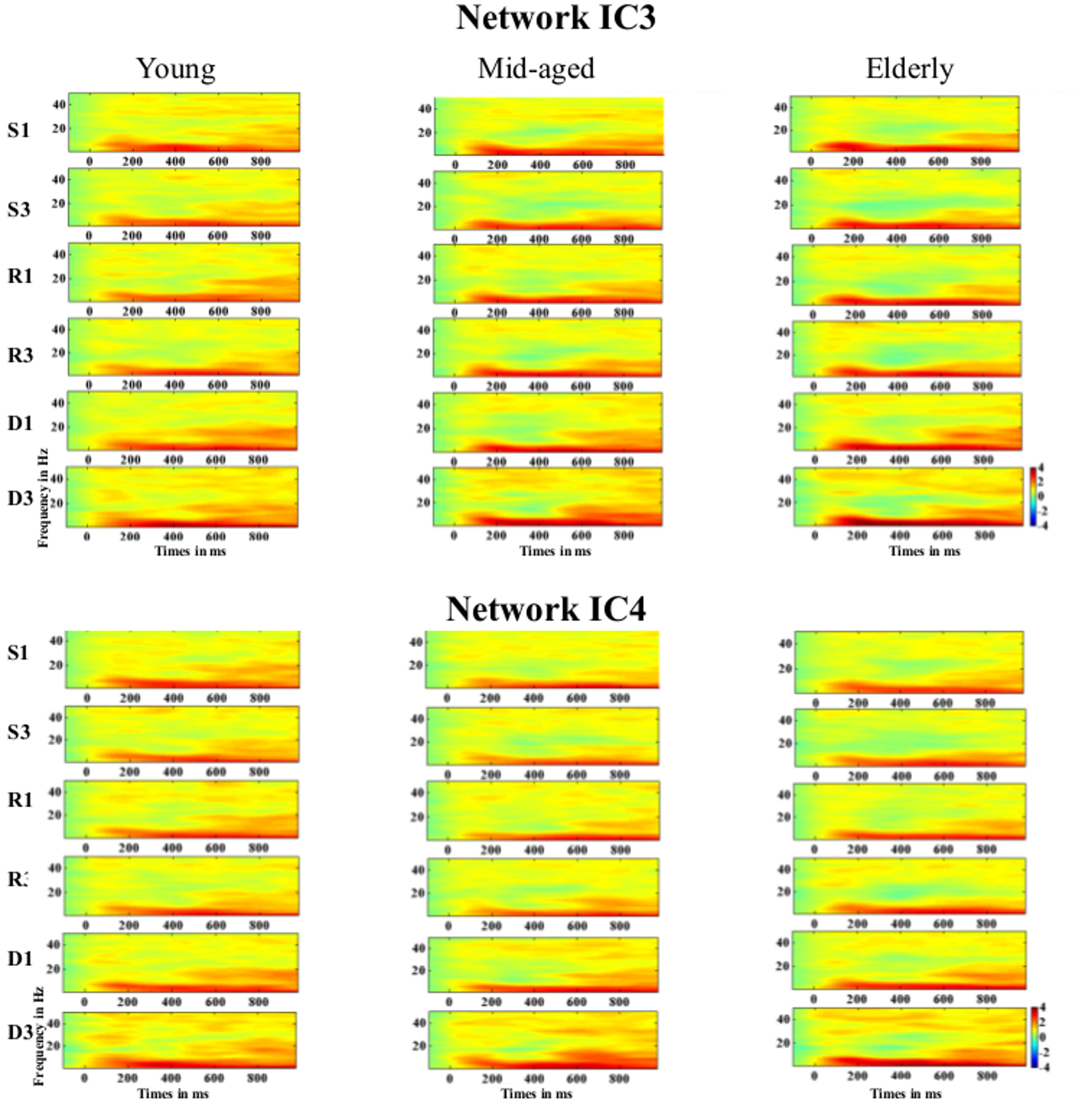

Supplement: Supplementary file 2 — Supplementary Figure 2. Independent component features of IC3 and IC4. Shown are the time-frequency plots (ERSP) of all conditions (S1: 1st Switch; S3: 3rd Switch; R1: 1st Repeat; R3: 3rd Repeat; D1: 1st Distractor; D3: 3rd Distractor) for the specific group and for all frequency bands (delta, beta, theta, alpha, low- and high gamma) for all age groups (young, mid-aged, elderly) of each component respectively. (TIFF 11510 kb) [file 10548_2016_512_MOESM2_ESM.tif]
